# New Fe_3_O_4_-Based Coatings with Enhanced Anti-Biofilm Activity for Medical Devices

**DOI:** 10.3390/antibiotics13070631

**Published:** 2024-07-07

**Authors:** Ioana Adelina Pirușcă, Paul Cătălin Balaure, Valentina Grumezescu, Stefan-Andrei Irimiciuc, Ovidiu-Cristian Oprea, Alexandra Cătălina Bîrcă, Bogdan Vasile, Alina Maria Holban, Ionela C. Voinea, Miruna S. Stan, Roxana Trușcă, Alexandru Mihai Grumezescu, George-Alexandru Croitoru

**Affiliations:** 1 Department of Science and Engineering of Oxide Materials and Nanomaterials, National University of Science and Technology POLITEHNICA Bucharest, 011061 Bucharest, Romania; adelina.pirusca@stud.fim.upb.ro (I.A.P.); ada_birca@yahoo.com (A.C.B.); bogdan.vasile@upb.ro (B.V.); truscaroxana@yahoo.com (R.T.);; 2 Department of Organic Chemistry, National University of Science and Technology POLITEHNICA Bucharest, 011061 Bucharest, Romania; 3 Lasers Department, National Institute for Laser, Plasma and Radiation Physics, 077125 Magurele, Romania; valentina.grumezescu@inflpr.ro (V.G.);; 4 Department of Inorganic Chemistry, Physical Chemistry and Electrochemistry, National University of Science and Technology POLITEHNICA Bucharest, 011061 Bucharest, Romania; ovidiu73@yahoo.com; 5 Microbiology and Immunology Department, Faculty of Biology, University of Bucharest, 77206 Bucharest, Romania; alina.holban@bio.unibuc.ro; 6 Research Institute of the University of Bucharest—ICUB, University of Bucharest, 050663 Bucharest, Romania; ionela-cristina.voinea@bio.unibuc.ro (I.C.V.); miruna.stan@bio.unibuc.ro (M.S.S.); 7 Department of Biochemistry and Molecular Biology, Faculty of Biology, University of Bucharest, 050095 Bucharest, Romania; 8 Department II, Faculty of Dental Medicine, Carol Davila University of Medicine and Pharmacy, 050474 Bucharest, Romania; alex.croitoru@umfcd.ro

**Keywords:** nosocomial infections, coatings, hydrophobic nanoparticles, usnic acid, ceftriaxone, sodium lauryl sulfate, biofilm inhibition

## Abstract

With the increasing use of invasive, interventional, indwelling, and implanted medical devices, healthcare-associated infections caused by pathogenic biofilms have become a major cause of morbidity and mortality. Herein, we present the fabrication, characterization, and *in vitro* evaluation of biocompatibility and anti-biofilm properties of new coatings based on Fe_3_O_4_ nanoparticles (NPs) loaded with usnic acid (UA) and ceftriaxone (CEF). Sodium lauryl sulfate (SLS) was employed as a stabilizer and modulator of the polarity, dispersibility, shape, and anti-biofilm properties of the magnetite nanoparticles. The resulting Fe_3_O_4_ functionalized NPs, namely Fe_3_O_4_@SLS, Fe_3_O_4_@SLS/UA, and Fe_3_O_4_@SLS/CEF, respectively, were prepared by co-precipitation method and fully characterized by XRD, TEM, SAED, SEM, FTIR, and TGA. They were further used to produce nanostructured coatings by matrix-assisted pulsed laser evaporation (MAPLE) technique. The biocompatibility of the coatings was assessed by measuring the cell viability, lactate dehydrogenase release, and nitric oxide level in the culture medium and by evaluating the actin cytoskeleton morphology of murine pre-osteoblasts. All prepared nanostructured coatings exhibited good biocompatibility. Biofilm growth inhibition ability was tested at 24 h and 48 h against *Staphylococcus aureus* and *Pseudomonas aeruginosa* as representative models for Gram-positive and Gram-negative bacteria. The coatings demonstrated good biocompatibility, promoting osteoblast adhesion, migration, and growth without significant impact on cell viability or morphology, highlighting their potential for developing safe and effective antibacterial surfaces.

## 1. Introduction

Pathogenic biofilms are at the root of the chronic and recurrent nature of many nosocomial infections. Bacteria can form biofilms on abiotic and biotic surfaces such as medical devices, hospital premise plumbing, hospital furniture, and human tissues, respectively. The European Centre for Disease Control (ECDC) estimates that 3.1–4.6 million people acquire a healthcare-associated infection in acute care hospitals in European Union (EU) and European Economic Area (EEA) countries combined, resulting in more than 90,000 deaths each year [1]. Therefore, it is crucially important to prevent the initial settlement by bacteria on abiotic or biotic surfaces and subsequent biofilm development [2,3]. 

Bacteria adhesion on surfaces is a very complex process resulting from a delicate interplay between diverse surface parameters such as surface topography, namely surface roughness and micropores; physical properties of the substrate like mechanical stiffness and wettability based on the molecular hydrophilic/lipophilic balance; and chemical properties of the surface, including surface energy, surface charge, and the presence of bioactive molecules on the substrate [4]. Wei et al. studied the adhesion of mouse fibroblast cells on hexamethyldisiloxane surfaces with a wide range of wettability obtained by varying the exposure periods to O_2_ plasma treatment, which introduces hydrophilic groups like COOH and OH [5]. The authors showed that cells spread more widely on the hydrophilic surfaces than on the hydrophobic surfaces in the initial stage and that super-hydrophilic surfaces (water contact angle approaching 0 degrees) were more favorable to cell attachment. Cheng et al. reported that a self-assembled monolayer (SAM) of 1-dodecanethiol on a gold substrate showed very low adhesion of *Pseudomonas aeruginosa* and ascribed this effect to the low surface energy of the methyl group [6]. 

Superparamagnetic iron oxide nanoparticles (SPIONs) have received much attention due to a series of interesting characteristics: intrinsic magnetic properties endowing them with magnetically targeted drug delivery and hyperthermia-induced drug release abilities, ease of synthesis and surface functionalization with bioactive molecules or active targeting ligands, biocompatibility, and *in vivo* biodegradability [7,8,9,10]. Magnetite (Fe_3_O_4_)-based theranostic agents show great promise toward advancing personalized cancer management [11,12,13]. Moreover, SPIONs present antimicrobial activity by themselves. They act as effective enzyme mimetics (nanozymes) with behavior similar to horseradish peroxidase; by reacting with hydrogen peroxide, they generate free radicals, namely reactive oxygen species (ROS), which are highly toxic to bacteria because they attack cell membranes by lipid peroxidation, and damage DNA and proteins [14,15].

**Figure 1:**
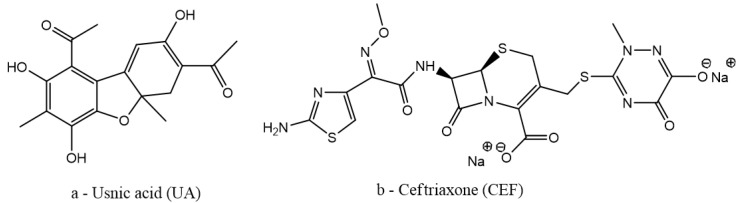
Chemical structures of the antimicrobial agents.

Usnic acid (UA), shown in Figure 1a, is a hydrophobic lichen secondary metabolite that was found to be able to inhibit biofilm formation [16,17,18,19,20,21,22,23].

Ceftriaxone (CEF), shown in Figure 1b, is a third-generation cephalosporin antibiotic that can bind to bovine serum albumin (BSA) in a process driven by hydrophobic interactions as a thermodynamic analysis of the parameters of the binding reaction showed [24].

Herein, we present the synthesis, physicochemical characterization, and *in vitro* evaluation of biocompatibility and antibiofilm properties of new coatings based on magnetite NPs having the following morphology: a magnetic iron oxide core surrounded by an inner nonpolar shell of SLS and an outer shell of adsorbed antimicrobial agent (UA or CEF). We chose this morphology with an inner nonpolar shell for three main reasons: First, this shell stabilizes the hydrophobic Fe_3_O_4_ NPs, which naturally tend to aggregate; second, the nonpolar inner shell can enhance attachment of the antimicrobial outer shell through hydrophobic interactions, and third, the outer shell will kill some surrounding bacteria, leaving behind the inner hydrophobic shell, which hopefully may impede the attachment of other still viable bacteria present in the environment.

The co-precipitation method was used to synthesize the core/shell Fe_3_O_4_@SLS NPs, which were further functionalized by adding the antimicrobial agent extra shell. The obtained nanopowders were then deposited as coatings by matrix-assisted pulsed laser evaporation (MAPLE), a deposition technique that provides a gentle process for fabricating coatings of small- and large-molecular-weight species and even thermally labile species such as organic compounds, polymers, and biomaterials, avoiding damaging by the high energy laser beam [25]. 

## 2. Results

### 2.1. Physicochemical Characterization of Magnetite Nanoparticles

**Figure 2:**
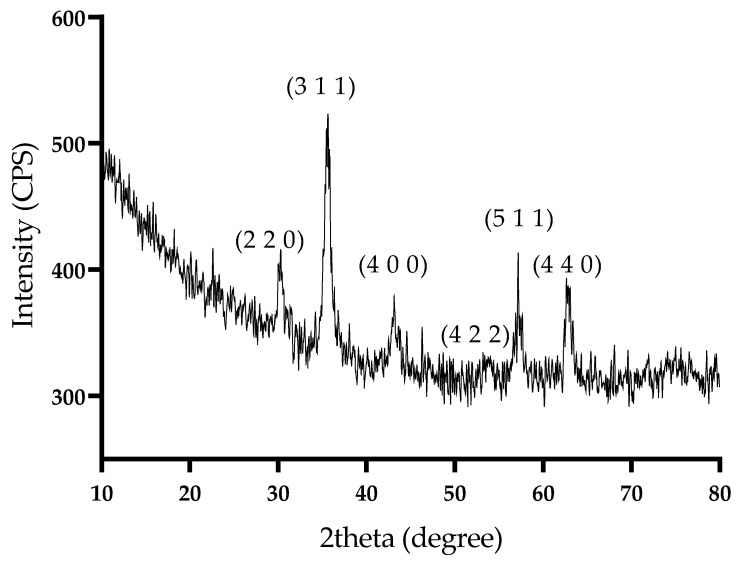
XRD diffractogram of Fe_3_O_4_@SLS.

X-ray diffraction (XRD) was used to identify crystalline phases present in the prepared NPs. Figure 2 depicts the diffractogram of Fe_3_O_4_@SLS. The sharp diffraction peaks observed at 2θ = 30.31, 35.71, 43.31, 53.90, 57.61, and 62.81 due to scattering from a specific set of parallel planes of atoms characterized by the Miller indices (2 2 0), (3 1 1), (4 0 0), (4 2 2), (5 1 1), and (4 4 0), respectively, are in very good agreement with those characteristic for magnetite, as reported by the American Society for Testing Materials (ASTM) sheet reference code 01-075-160 [26]. Moreover, no other diffraction peaks corresponding to another iron oxide, such as α-Fe_2_O_3_ or γ-Fe_2_O_3_, could be observed. The average crystallite size was 19.8 nm, and it was determined with the Debye–Scherrer formula.

**Figure 3:**
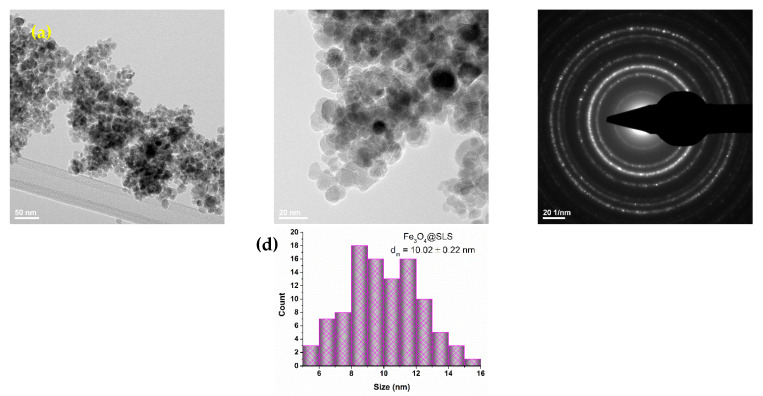
TEM (**a**,**b**) micrographs, SAED pattern (**c**), and size distribution (**d**) of Fe_3_O_4_@SLS NPs.

A TEM micrograph of Fe_3_O_4_@SLS is depicted in Figure 3, highlighting areas with dispersed nanoparticles as well as areas of homo-aggregates. The recorded SAED ring pattern of Fe_3_O_4_@SLS plotted in Figure 3c corresponds to the (2 2 0), (3 1 1), (4 0 0), (4 2 2), (5 1 1), and (4 4 0) magnetite lattice planes and confirms the presence of a single one-crystalline magnetite phase, which is in good agreement with the results of the XRD analysis.

**Figure 4:**
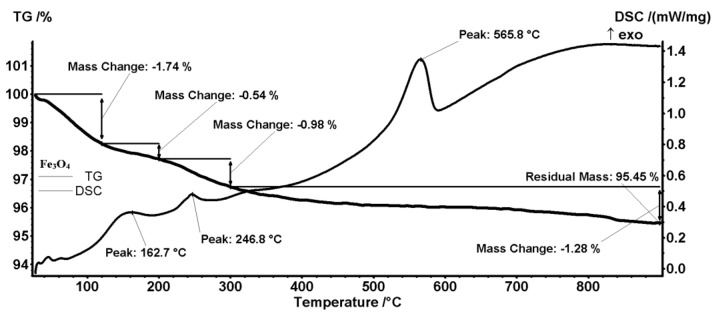
Combined TGA-DSC thermogram of pristine Fe_3_O_4_ NPs.

**Figure 5:**
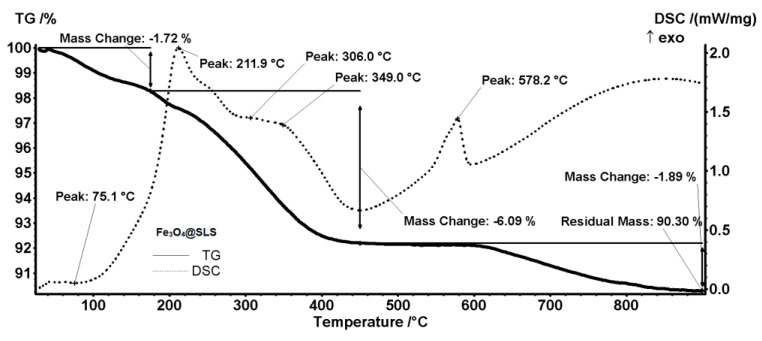
Combined TGA-DSC thermogram of core/shell Fe_3_O_4_@SLS NPs.

Temperature-driven changes in the prepared NPs were studied by means of combined thermogravimetric analysis (TGA) and differential scanning calorimetry (DSC). The TGA-DSC thermograms in Figure 4 and Figure 5 correspond to pristine Fe_3_O_4_ and core/shell Fe_3_O_4_@SLS NPs, respectively. Figure 4 shows that the first mass loss of 1.74% occurs between room temperature and 120 °C due to magnetite dehydration in a slightly endothermic process. Next, two successive mass losses of 0.54% and 0.98% occur in the temperature ranges 120–200 °C and 200–300 °C, being marked on the DSC curve by the apparent peaks at 162.7 °C and 246.8 °C, respectively. These mass losses are caused by the exothermic oxidation of some organic impurities present on the magnetite surface. Eventually, the strong exothermic peak at 565.8 °C is related to the phase transition to maghemite (γ-Fe_2_O_3_) [27]. The mass loss of the latter transformation is 1.28%. Thus, the total mass loss is 4.54%, corresponding to a residual mass of 95.45% wt. of the initial mass. Similar mass losses and enthalpy changes can be seen on the TGA-DSC thermogram in Figure 5. Dehydration and loss of -OH groups from magnetite’s surface (1.72%) occurs from room temperature up to 180 °C. The next two endothermic mass losses (6.09% and 1.89%) observed between 440–480 °C, and 800–900 °C, respectively, correspond to the oxidation of organic materials, while the sharp exothermic peak at 578.2 °C marks the phase transition from magnetite to maghemite. The residual mass is 90.3%. Assuming that the organic shell of SLS was completely degraded during the analysis, a simple calculation gives a rough estimate of the SLS coating weight, namely 5.39 g of SLS/100 g of magnetite.

### 2.2. Physicochemical Characterization of the Coatings

**Figure 6:**
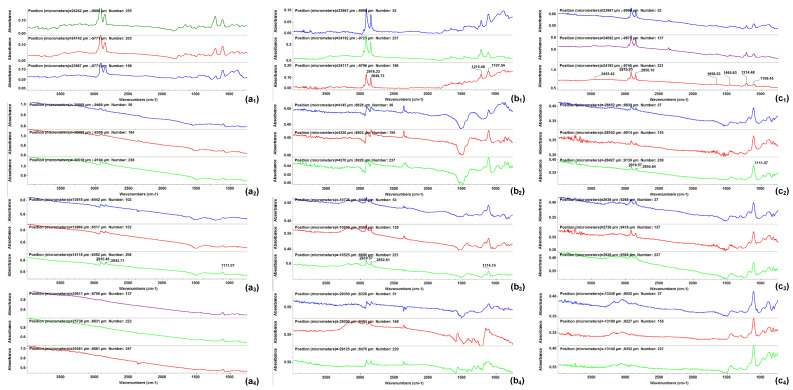
FT-IR spectra of (**a_1_**–**a_4_**) Fe_3_O_4_@SLS, (**b_1_**–**b_4_**) Fe_3_O_4_@SLS/UA, and (**c_1_**–**c_4_**) Fe_3_O_4_@SLS/CEF at (**a_2_**,**b_2_**,**c_2_**) F = 200 mJ/cm^2^, (**a_3_**,**b_3_**,**c_3_**) F = 300 mJ/cm^2^, and (**a_4_**,**b_4_**,**c_4_**) F = 400 mJ/cm^2^ laser fluences and (**a_1_**,**b_1_**,**c_1_**) dropcast.

In order to determine the best compromise between the deposition efficiency and compositional integrity of the obtained coatings, comparative infrared studies were conducted between the dropcast sample and coatings (Figure 6). The surface distribution of the signal intensity of specific spectral markers was monitored by IR microscopy. In order to plot the intensities of spectral IR absorptions characterizing specific functional groups (spectral markers) in correspondence with their spatial position in a selected area, chemical maps were generated [28,29,30]. A chromatic scale is used to code the absorption intensities of specific spectral markers. The predominance of the blue color indicates a low intensity of the monitored IR absorption, which is associated with a low amount of deposited material. On the contrary, areas with high intensities of the monitored IR absorptions appear colored in red. 

**Figure 7:**
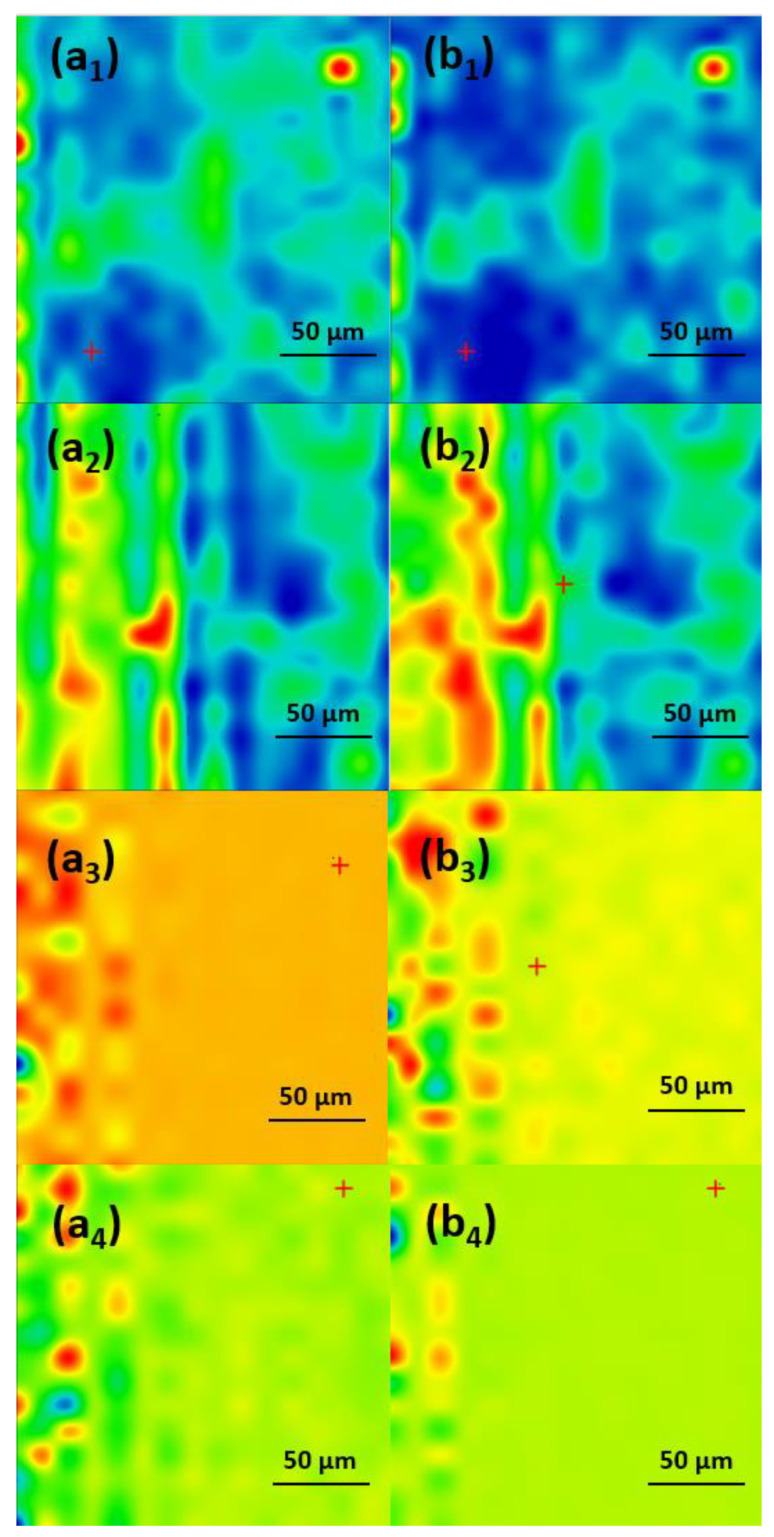
IR maps of Fe_3_O_4_@SLS created based on –CH_2_– and S–O groups at surface intensity distributions of (**a_1_**–**a_4_**) 2916 cm^−1^ and (**b_1_**–**b_4_**) 1107 cm^−1^ for (**a_1_**,**b_1_**) dropcast and coatings deposited at (**a_2_**,**b_2_**) 200 mJ/cm^2^, (**a_3_**,**b_3_**) 300 mJ/cm^2^, and (**a_4_**,**b_4_**) 400 mJ/cm^2^ laser fluences.

**Figure 8:**
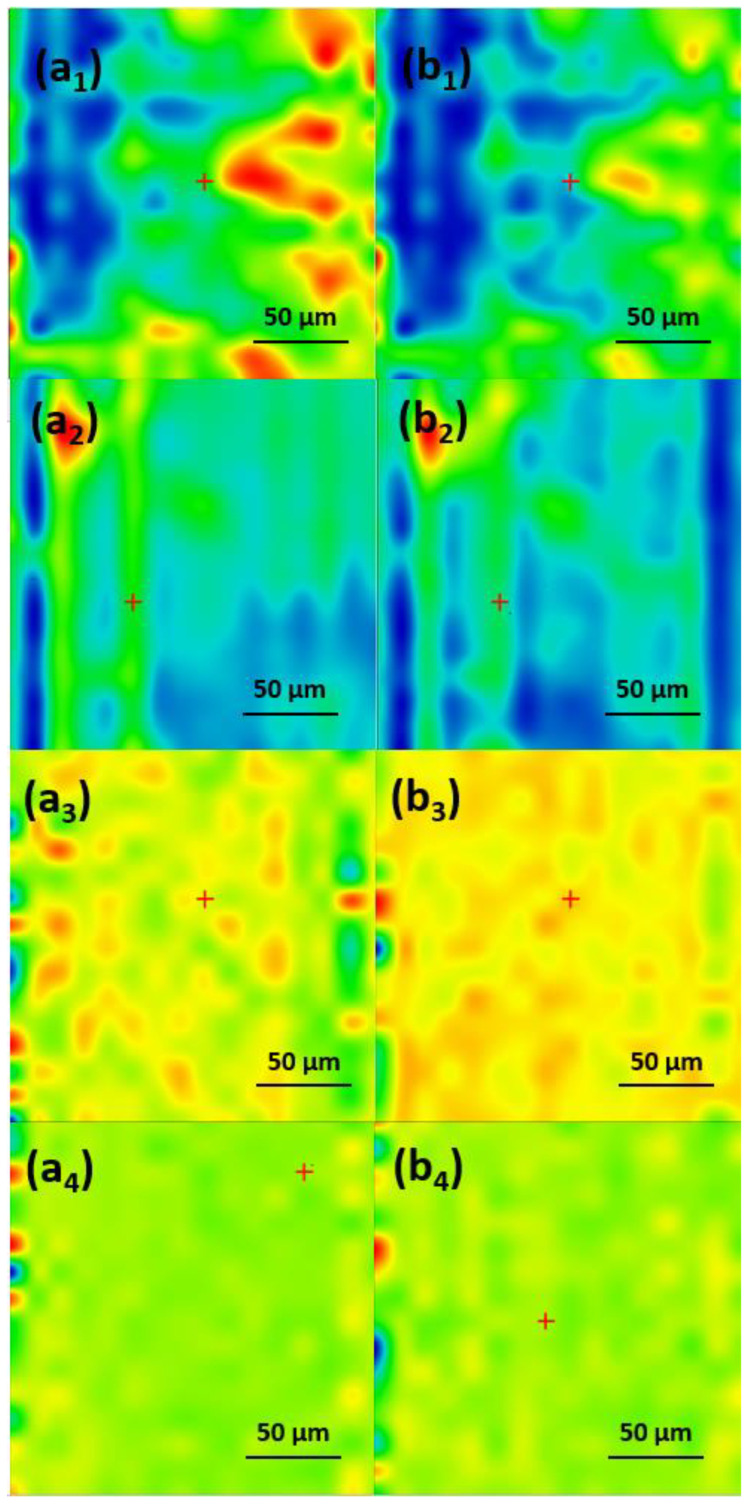
IR maps of Fe_3_O_4_@SLS/UA created based on C=O and S–O groups at surface intensity distributions of (**a_1_**–**a_4_**) 1697 cm^−1^ and (**b_1_**–**b_4_**) is 1107 cm^−1^ for (**a_1_**,**b_1_**) dropcast and coatings deposited at (**a_2_**,**b_2_**) 200 mJ/cm^2^, (**a_3_**,**b_3_**) 300 mJ/cm^2^, and (**a_4_**,**b_4_**) 400 mJ/cm^2^ laser fluences.

**Figure 9:**
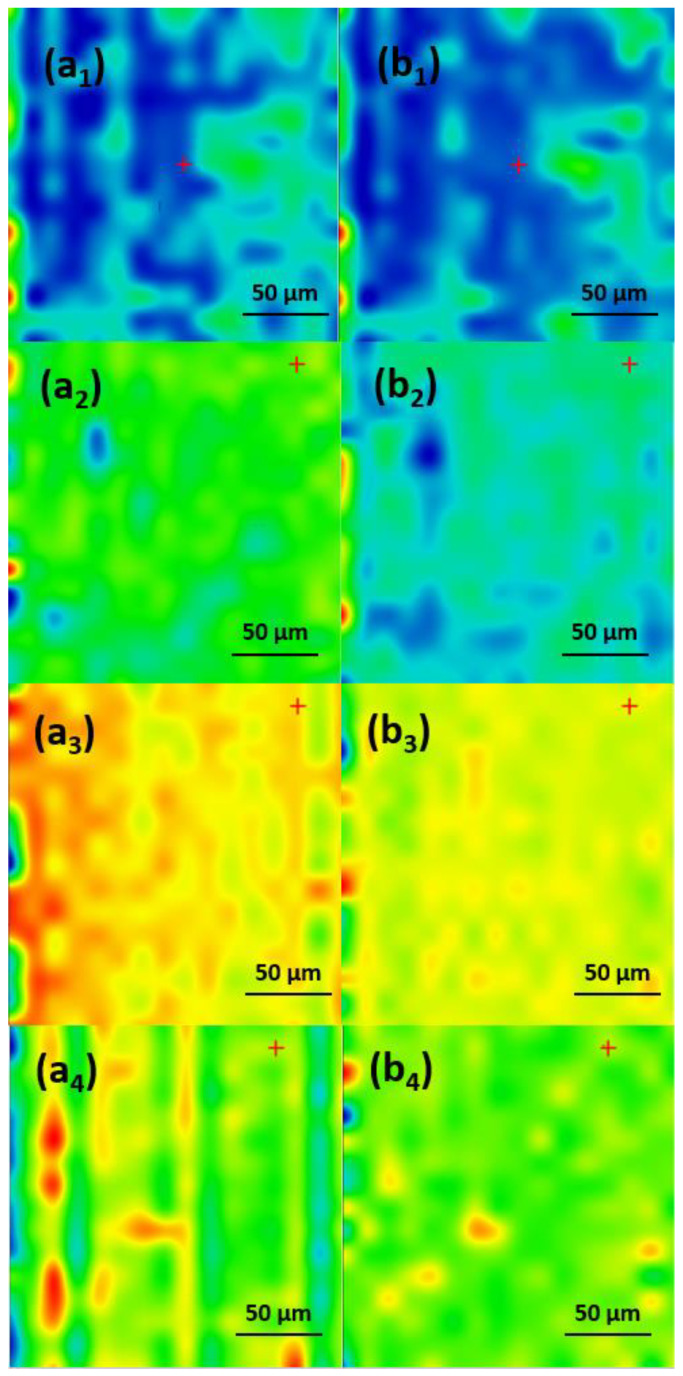
IR maps of Fe_3_O_4_@SLS/CEF created based on C=O and S–O groups at surface intensity distributions of (**a_1_**–**a_4_**) 1658 cm^−1^ and (**b_1_**–**b_4_**) 1107 cm^−1^ for (**a_1_**,**b_1_**) dropcast and coatings deposited at (**a_2_**,**b_2_**) 200 mJ/cm^2^, (**a_3_**,**b_3_**) 300 mJ/cm^2^, and (**a_4_**,**b_4_**) 400 mJ/cm^2^ laser fluences.

The presence of monitored IR absorptions can be observed at ~2916 cm^−1^ associated with CH_2_ stretching, while the peak at ~1107 cm^−1^ is characteristic of SO_4_^−^- stretching, indicative of the SLS nonpolar inner shell. The peak at ~1697 cm^−1^ corresponds to the stretching vibration of the carbonyl C=O group characteristic for the UA (outer extra shell), and ~1658 cm^−1^ corresponds to the amidic carbonyl group in CEF. These IR bands were used to generate the IR maps (Figure 7, Figure 8 and Figure 9) [31,32,33,34,35].

As one can observe from the comparative analysis of IRM micrographs (Figure 7, Figure 8 and Figure 9), the structural integrity was maintained at 300 mJ/cm^2^ laser fluence. In the case of the coatings obtained at 200 mJ/cm^2^, the predominance of the blue color indicates a low deposition rate (Figure 7(a_2_,b_2_)).

The red color corresponding to the IR maps at 300 mJ/cm^2^ (Figure 7(a_3_,b_3_)) indicates a high abundance of the monitored functional groups, which can be being associated with a high deposition rate. The green color present at 400 mJ/cm^2^ (Figure 7(a_4_,b_4_)) corresponds to an intermediate intensity of the monitored IR absorptions, indicating a significant degradation of the functional groups in the deposited material due to the higher energy of the laser beam. Similar results were obtained for the coatings with an extra shell of antimicrobial agents, i.e., Fe_3_O_4_@SLS/UA (Figure 8) and Fe_3_O_4_@SLS/CEF (Figure 9), respectively. Based on the results provided by IR analysis, the following investigations were performed only on Fe_3_O_4_@SLS materials processed at 300 mJ/cm^2^ laser fluence.

**Figure 10:**
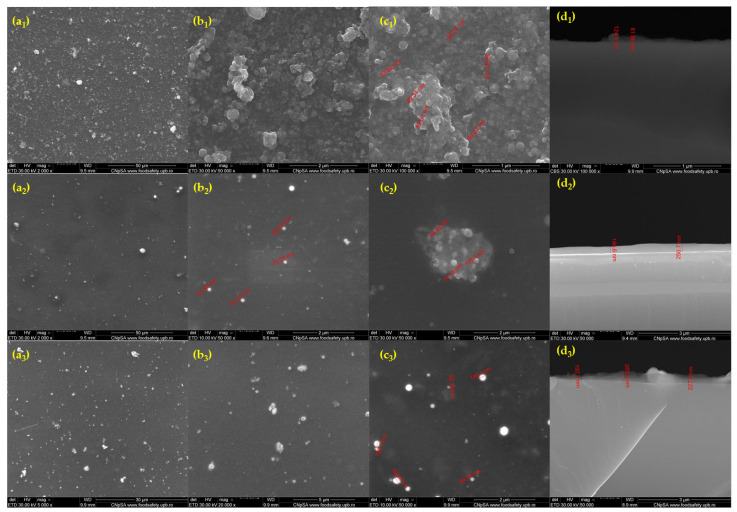
SEM micrographs of Fe_3_O_4_@SLS (**a1**,**b1**,**c1**,**d1**), Fe_3_O_4_@SLS/UA (**a2**,**b2**,**c2**,**d2**), and Fe_3_O_4_@SLS/CEF (**a3**,**b3**,**c3**,**d3**) plan-view (**a1**–**a3**,**b1**–**b3**,**c1**–**c3**) and cross-sections (**d1**–**d3**) recorded at various magnifications.

Relevant microstructural information was obtained by both top-view and cross-section SEM micrographs (Figure 10). Under high magnification, the formation of clusters of magnetite NPs with relatively good dispersibility can be seen. SEM images reveal a particle size distribution in the range of 25–60 nm for Fe_3_O_4_@SLS, in the range of 45–100 for Fe_3_O_4_@SLS/UA, and in the range of 55–200 for Fe_3_O_4_@SLS/CEF. The thickness of the deposited coating ranges from 80 to 125 nm for Fe_3_O_4_@SLS, 180 to 250 nm for Fe_3_O_4_@SLS/UA, and 190 to 295 nm for Fe_3_O_4_@SLS/CEF. The continuous (sub-)micron coatings were composed of particulates of different sizes and arbitrarily scattered on the surface, which had a positive outcome on cell adhesion and proliferation.

### 2.3. Biological Evaluation

#### 2.3.1. Biocompatibility on Pre-Osteoblasts

**Figure 11:**
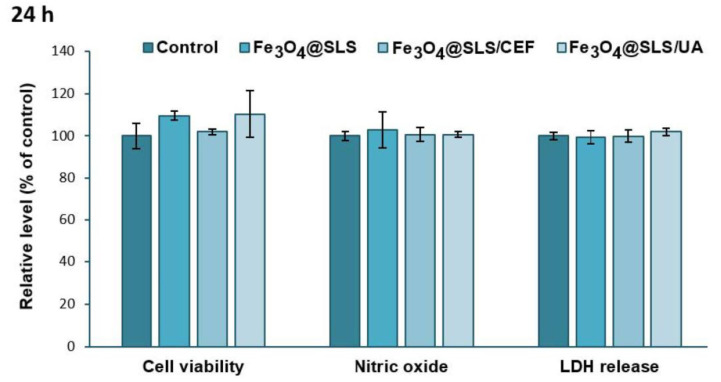
Biocompatibility of Fe_3_O_4_@SLS, Fe_3_O_4_@SLS/CEF, and Fe_3_O_4_@SLS/UA coatings, as shown by cell viability (MTT assay), NO level, and LDH release after 24 h of exposure on MC3T3-E1 murine cells. All results were calculated as mean ± standard deviations of three different replicates and expressed relative to control.

**Figure 12:**
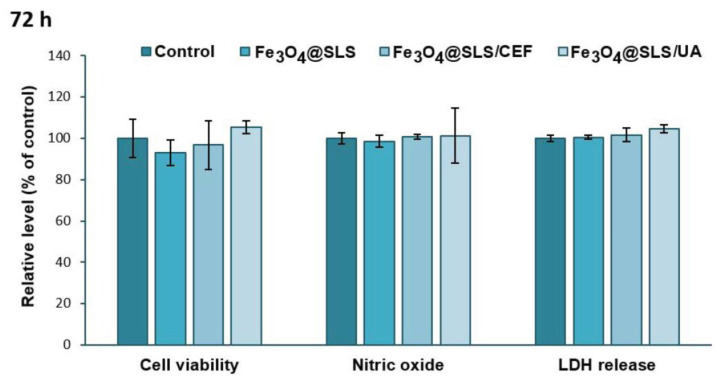
Biocompatibility of Fe_3_O_4_@SLS, Fe_3_O_4_@SLS/CEF, and Fe_3_O_4_@SLS/UA coatings, as shown by cell viability (MTT assay), NO level, and LDH release after 72 h of exposure on MC3T3-E1 murine cells. All results were calculated as mean ± standard deviations of three different replicates and expressed relative to control.

Assessment of the cytotoxic effects exerted on eukaryotic cells is necessary every time the development of new and biocompatible antibacterial agents is desired. Therefore, the biological response of Fe_3_O_4_@SLS, Fe_3_O_4_@SLS/CEF, and Fe_3_O_4_@SLS/UA coatings with anti-biofilm potential was evaluated on pre-osteoblast murine cells. As shown in Figure 11 and Figure 12, the exposure of MC3T3-E1 cells to the modified glass surfaces for 24 and 72 h did not induce significant changes in cell viability, NO level, and LDH release, suggesting that these surfaces presented great biocompatibility without inducing inflammatory processes or affecting the integrity of cell membranes.

**Figure 13:**
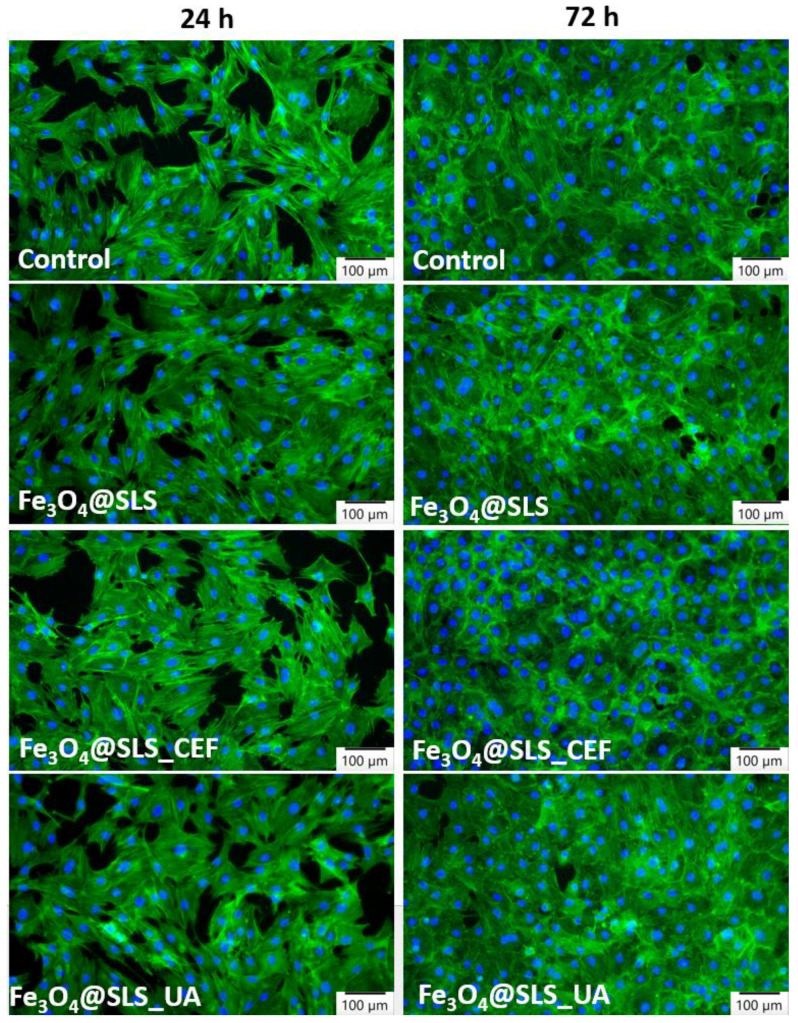
Actin cytoskeleton organization of MC3T3-E1 murine cells after 24 and 72 h of incubation with Fe_3_O_4_@SLS, Fe_3_O_4_@SLS/CEF, and Fe_3_O_4_@SLS/UA coatings. F-actin (green) was labeled with phalloidin-fluorescein isothiocyanate (FITC), and nuclei (blue) were counterstained with 4′,6-diamidino-2-phenylindole dihydrochloride (DAPI). Magnification (20× objective).

In order to assess the effect of SPIONs-based anti-biofilm coatings on cell morphology, actin cytoskeleton organization was investigated by fluorescence microscopy (Figure 13). MC3T3-E1 cell behavior was not significantly influenced in the presence of these coatings compared to the control, and these results are in agreement with the biocompatibility assays. The nanostructured surfaces obtained by MAPLE technique successfully promoted cell adhesion, migration, and growth.

#### 2.3.2. Effects on Pathogenic Biofilms Growth

**Figure 14:**
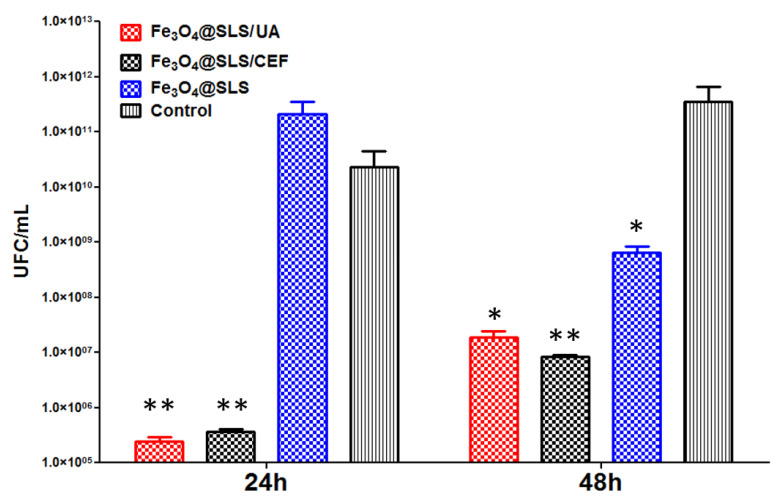
Evaluation of *S. aureus* biofilm growth after 24/48 h of incubation with Fe_3_O_4_@SLS, Fe_3_O_4_@SLS/CEF, and Fe_3_O_4_@SLS/UA coatings vs. control (one-way ANOVA, when comparing samples vs. control * *p* < 0.05; ** *p* < 0.001).

**Figure 15:**
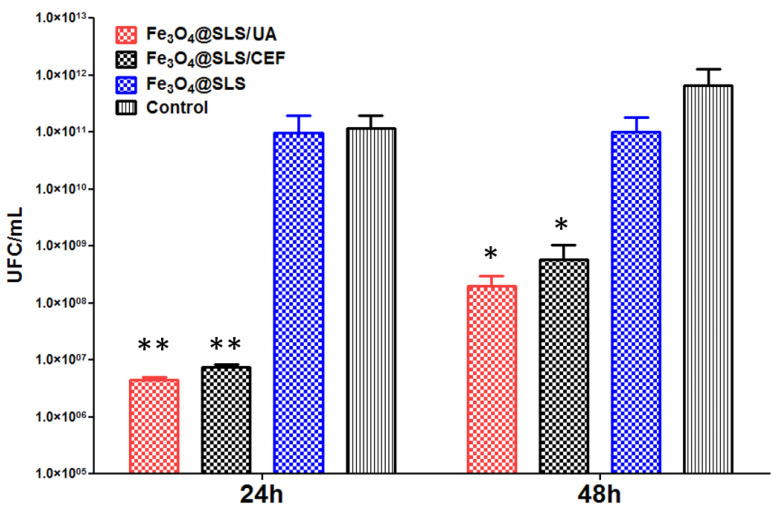
Evaluation of *P. aeruginosa* biofilm growth after 24/48 h of incubation with Fe_3_O_4_@SLS, Fe_3_O_4_@SLS/CEF, and Fe_3_O_4_@SLS/UA coatings vs. control (one-way ANOVA, when comparing samples vs. control * *p* < 0.05; ** *p* < 0.001).

Figure 14 and Figure 15 below reveal the ability of nano-modified surfaces to alter the development of bacterial biofilms at different time intervals (24 and 48 h) against Gram-negative (*P. aeruginosa*) and Gram-positive (*S. aureus*) opportunistic pathogens.

The nanocoated samples determined an important decrease in the bacterial populations of *S. aureus* (Figure 14) and *P. aeruginosa* (Figure 15) as compared to the uncoated control specimens. Little modification in the CFU/mL values was also observed when comparing control samples with the Fe_3_O_4_@SLS coating. The anti-biofilm effect of the Fe_3_O_4_@SLS control sample was not present in *S. aureus* monospecific biofilms after 24 h of incubation, but it started to show after 48 h.

A significant decrease in the *S. aureus* CFU/mL biofilm values was observed for the samples coated with Fe_3_O_4_@SLS/CEF and Fe_3_O_4_@SLS/UA, where the number of viable biofilm cells was reduced by up to 4 logs. The biofilm inhibition potential of both coatings was maintained for at least 48 h in the evaluated conditions.

Further, a comparable and sustained inhibition of biofilms was noticed also for the tested Gram-negative bacteria, namely *P. aeruginosa*. Fe_3_O_4_@SLS/CEF and Fe_3_O_4_@SLS/UA reduced the *P. aeruginosa* monospecific biofilm development by 3.5 logs at 24 h of incubation and by 2 logs after 48 h of contact (Figure 15).

Although both variants of antimicrobial compounds are efficient in reducing the microbial biofilm development when they are present in deposited coatings on medical surfaces, the coating containing UA seems more efficient in biofilm inhibition, and this correlates with the great antimicrobial effect of this natural compounds demonstrated especially in Gram-positive species [36].

## 3. Discussion

Fe_3_O_4_@SLS, Fe_3_O_4_@SLS/CEF, and Fe_3_O_4_@SLS/UA MAPLE-deposited coatings are highly biocompatible without significant damage of the eukaryotic plasma membrane and with a lack of any inflammatory response, as fully demonstrated by the MTT cell viability, LDH leakage, and NO inflammatory marker tests carried out on MC3T3-E1 pre-osteoblast murine cells. These results were confirmed by the fluorescence microscopy studies, revealing no important alterations of the normal cell morphology and actin cytoskeleton organization.

Furthermore, the results presented herein demonstrate that our morphological design of the prepared NPs with a magnetite core surrounded by an inner surfactant (SLS) shell and an outer antibiotic (CEF) or lichen secondary metabolite (UA) shell succeeded in synergistically increasing the antimicrobial and anti-biofilm activities. There are a few previous studies reporting on the capacity of the anionic surfactant SLS not only to prevent bacteria adhesion to hydrophobic surfaces and the onset of biofilm formation but also to promote bacterial detachment [37,38,39,40,41,42,43,44,45]. SDS can alter microbial cells and biofilms in multiple ways. Due to its amphiphilic nature, it causes protein unfolding, thereby promoting biofilm detachment through denaturation of proteinaceous matrix adhesions [40]. It can also kill planktonic bacteria by penetrating the cytoplasmic membrane and causing cell lysis [40]. SDS is able to disrupt bacterial cell-to-cell communications through pili and nanotubes, thereby impeding biofilm development as well as horizontal gene transfer and conjugational spread of virulence factors [41,42,43]. Diaz De Rienzo et al. demonstrated that in mixture with caprylic acid, SDS inhibited the growth of foodborne pathogens *Bacillus subtilis* NCTC 10400 and *S. aureus* [44]. Biofilms formed by an isogenic and poly-*N*-acetyl-glucosamine-deficient mutant strain of *Aggregatibacter actinomycetemcomitans*, which is the pathogenic agent responsible for several forms of destructive periodontal disease, were found susceptible to detachment by SDS, as demonstrated by Izano et al. [40]. Li et al. developed an antibiofilm coating based on the controlled release of SLS from a nanoporous, hydrophobic, and non-biodegradable 1,2-polybutadiene (PB) matrix [45]. The fabrication of the nanoporous matrix started with a diblock copolymer precursor, namely 1,2-(PB)-*b*-polydimethylsiloxane (PDMS). The PB block was cross-linked in tetrahydrofuran (THF) solution at 140 °C under a nitrogen atmosphere for 2 h using dicumyl peroxide (DCP) as a cross-linker when the diblock copolymer self-assembled into a gyroid morphology. Subsequently, the PDMS block was quantitatively removed by chemical etching with a THF solution of tetra-*n*-butylammonium fluoride (TBAF) at room temperature for 36 h. The nucleophilic attack of the fluoride ion on the siloxane Si–O bond resulted in the disintegration of the covalent backbone of the PDMS block. The monomeric fragments were extracted by the organic solvent, leaving behind a nanoporous matrix of 1,2(PB), which eventually was filled up with SLS. The SLS-loaded nanoporous PB coating was proved to block the attachment of *E. coli* in the short term (3 h), while significantly reducing biofilm formation in the long term (1 week). By tuning the morphologic characteristics of the nanoporous polymeric matrix, the release profile of SLS could be controlled, and the anti-biofilm activity of the nanocoating was extended [45].

Regarding our results presented here, as one can notice in Figure 14 and Figure 15, after incubation for 48 h with the bacterial cultures, Fe_3_O_4_@SLS nanoparticles showed a slight inhibition of the biofilm growth, as indicated by the drop observed in the value of CFU/mL, with the effect being stronger for the *S*. *aureus* pathogen. Normally, the addition of the antimicrobial agent (CEF/UA) extra shell gave a powerful boost to the biofilm inhibition effect, marked by a dramatic drop by 4–5 orders of magnitude in the CFU/mL value versus the corresponding value for the untreated control in the short term (24 h). The effect was manifested on both pathogens but was more intense in the case of *S*. *aureus* culture.

## 4. Materials and Methods

### 4.1. Materials

Analytical-grade reagents were purchased from Sigma-Aldrich/Merck (Darmstadt, Germany), and used without additional purification to synthesize the materials of interest.

### 4.2. Chemical Synthesis of Sodium Lauryl Sulfate Covered Magnetite Fe_3_O_4_@SLS Nanoparticles

In the synthesis of nonpolar functionalized magnetite NPs, two distinct solutions were prepared. Solution 1: In a 600 mL beaker, 300 mL of deionized water was used as a solvent in which 1.6 g of ferrous sulfate (FeSO_4_) and 1 g of ferric chloride (FeCl_3_) were sequentially dissolved. Each solute was stirred thoroughly using a glass rod after its addition. Solution 2: A separate 600 mL beaker was used to prepare another mixture consisting of 300 mL deionized water, 0.2 g of sodium lauryl sulfate (SLS), and 9 mL of ammonium hydroxide (NH_4_OH). This mixture was then subjected to magnetic stirring to ensure homogeneity.

Following the preparation of these solutions, solution 1 was added dropwise to solution 2 while continuously stirring on a magnetic stirrer. The magnetic particles were then washed three times with deionized water. Finally, the washed particles were left to dry at room temperature.

### 4.3. Chemical Syntheses of Magnetite NPs Functionalized with Antimicrobial Agents UA and CEF

For the synthesis of NPs functionalized with cephalosporin, the initial steps were identical to those described for the inorganic core preparation. For the second solution, however, 0.2 g of cephalosporin was added over SLS, followed by 9 mL of NH_4_OH. The mixture was then magnetically stirred.

Similarly, for NPs functionalized with usnic acid, the procedure mirrored that of the initial core synthesis, with the second solution comprising 0.2 g of UA over SLS, combined with 9 mL of NH_4_OH and subjected to magnetic stirring. After this, the synthesis process was identical to the one described in Section 2.2.

### 4.4. MAPLE Experimental Conditions for Obtained Coatings

A solution of 3% Fe_3_O_4_@SLS, 1% Fe_3_O_4_@SLS@UA, and Fe_3_O_4_@SLS@CEF dissolved in DMSO was poured into a pre-cooled target holder and subsequently immersed in liquid nitrogen for 30 min.

In the deposition chamber, these targets were maintained at liquid nitrogen temperature due to the cooling system and irradiated with a KrF* laser source (λ = 248 nm, τFWHM = 25 ns) from COMPexPro 205 (Lambda Physics-Coherent, Gottingen, Germany) that operated at a repetition rate of 15 Hz. All depositions were conducted at room temperature, maintaining a constant pressure of 0.1 Pa. The coatings were grown at a target–substrate separation distance of 4 cm by applying 80,000 subsequent laser pulses for each experiment. The energy distribution of the laser spot was improved by means of a laser beam homogenizer. The laser fluence was within the range of (200–400) mJ/cm^2^, whereas the laser spot area was set to 30 mm^2^. All the deposition was made on glass and Si (1 0 0) substrates. 

### 4.5. Physicochemical Characterization

An investigation of the crystallinity of the prepared core/shell magnetite NPs was carried out using an XRD analysis performed on a Shimadzu XRD 6000 diffractometer (Duisburg, Germany). Recordings of the X-ray diffraction patterns were conducted at room temperature with the Bragg diffraction angle 2θ ranging between 10 and 80° using CuKα radiation (λ = 1.54056 Å at 15 mA and 30 kV).

The structure and phase composition of the nanomaterials were observed by transmission electron microscopy (TEM). TEM micrographs were acquired on a TecnaiTM G2 F30 S-TWIN electron microscope equipped with selected area electron diffraction (SAED) purchased from the FEI (Hillsboro, OR, USA) company. The microscope was operated in transmission mode at 300 kV. The point and line resolutions of the TEM images were 2 Å and 1.02 Å, respectively. TEM specimens were obtained by dispersing the nanopowders in ethanol and ultrasonic cleansing for 15 min. Afterward, the sample was placed on a carbon-coated copper grid and left to dry at room temperature.

The surface morphology and size nanodetails of the prepared nanopowders and coatings were revealed by SEM analyses performed on an FEI electron microscope (Thermo Fisher, Eindhoven, The Netherlands), using secondary electron beams with energies of 30 keV. 

In order to determine the best compromise between the deposition efficiency and compositional integrity of the deposited coatings, the surface distribution of the signal intensity of specific spectral markers was monitored, namely absorptions characteristic of the chemical functional groups of the deposited material, i.e., C–H and S=O bonds in SLS and C=O bonds in UA and CEF, respectively. IR mapping (IRM) was performed on a Nicolet iN10 MX FT-IR microscope with an MCT liquid nitrogen-cooled detector, which operates in the range 4000–1000 cm^−1^. The FT-IR microscopy maps were acquired in the reflection mode at 4 cm^−1^ resolution. A total of 32 scans were co-added and converted to absorbance for each spectrum using Ominc Picta 8.2 software (Thermo Scientific, Waltham, MA, USA). About 250 spectra were analyzed for each sample.

The thermal analyses were assessed with Shimadzu DTG-TA-50H equipment (Carlsbad, CA, USA) from room temperature to 800 °C at a heating rate of 10 K min^−1^. All measurements were performed in the presence of dried synthetic air (80% N_2_ and 20% O_2_) purge (20 mL min^−1^).

### 4.6. Biological Tests

#### 4.6.1. Assessment of Biocompatibility on Pre-Osteoblast Cells

*In vitro* biological response to the anti-biofilm coatings was evaluated using MC3T3-E1 pre-osteoblast cell line (purchased from American Type Culture Collection—ATCC, Cat. No. CRL-2593, Rockville, MD, USA). The cells were grown to 70–80% confluence in Dulbecco’s Modified Eagle Medium (DMEM) without Phenol Red (Gibco/Invitrogen, Carlsbad, CA, USA) and supplemented with 10% *v*/*v* fetal bovine serum (FBS; Gibco/Invitrogen, Carlsbad, CA, USA) in a humidified atmosphere with 5% CO_2_ at 37 °C. For all the biological assays, cells were detached using trypsin/EDTA solution (0.25% *w*/*v* trypsin/0.02% EDTA), counted, and seeded at a density of 3 × 10^4^ cells/cm^2^ on sterile coated glass substrates into 24-well plates. Controls represented by uncoated glass substrates were also tested. After 24 h and 72 h of cell exposure to the modified surfaces, several biocompatibility tests were performed. Cell viability was assessed using the 3-(4,5-dimethylthiazol-2-yl)-2,5-diphenyltetrazolium bromide (MTT; Sigma-Aldrich, St. Louis, MO, USA) assay. Briefly, the culture medium was removed from each well, and cells were incubated with 1 mg/mL MTT solution for 2 h at 37 °C and 5% CO_2_ at the end of the exposure time. The resulting purple formazan crystals were dissolved with isopropanol (Sigma-Aldrich, St. Louis, MO, USA), and the absorbance was measured at 595 nm using a FlexStation 3 microplate reader (Molecular Devices, Sunnyvale, CA, USA). Cell membrane integrity was determined using a commercially available cytotoxicity assay kit (Cytotoxicity Detection Kit-LDH, Roche, Basel, Switzerland) according to the manufacturer’s instructions. In addition, the nitric oxide (NO) level in the culture medium was measured as an inflammatory marker using the Griess reagent. 

Actin cytoskeleton and cell morphology dynamics were visualized and investigated by immunofluorescence staining. At the end of each interval exposure, cells were fixed with 4% paraformaldehyde for 20 min and permeabilized with 0.1% Triton X-100/2% BSA for 1 h. Filamentous actin (F-actin) was stained with 20 μg/mL phalloidin conjugated with fluorescein isothiocyanate (FITC), and nuclei were counterstained with 2 μg/mL 4′,6-diamidino-2-phenylindole (DAPI) (Molecular Probes, Life Technologies, Carlsbad, CA, USA). Representative images for each tested surface were captured using a fluorescence microscope Olympus IX71 (Olympus, Tokyo, Japan).

#### 4.6.2. Anti-Biofilm Activity

For assessing the antibacterial efficiency of obtained coatings, we utilized monospecific biofilms developed by model opportunistic pathogens: the Gram-positive (*Staphylococcus aureus* ATCC^®^ 25923) and Gram-negative (*Pseudomonas aeruginosa* ATCC^®^ 27853) bacterial strains. The development of biofilms was assessed in the presence of MAPLE-coated surfaces for different time points (24 h and 48 h).

Briefly, the UV-sterilized control (uncoated) and nanocoated specimens were transferred to sterile 24-well plates containing 1 mL of Luria–Bertani (LB) broth (Thermo Fischer Scientific), then inoculated with 10 μL of 0.5 McFarland standard-density microbial suspensions (1.5 × 10^8^ CFU (colony forming units)/mL). The prepared plates were incubated at 37 °C for 24 h, then the culture media was removed, and the samples were washed with sterile phosphate-buffered saline (PBS, Sigma/Merck). Then, the samples were transferred to new sterile plates containing fresh LB broth and incubated at 37 °C for 24 h and 48 h. After incubation, samples were gently washed with PBS and transferred to 1.5 mL centrifuge tubes containing sterile PBS. All specimens were successively vortexed for 20 s and sonicated for 10 s to detach the biofilm cells and obtain cell suspensions. Serial ten-fold dilutions were further performed from the biofilm-embedded microbial cells, which were obtained as uniform suspensions, and the obtained PBS dilutions were then seeded on LB agar plates to evaluate the colony-forming units (CFU/mL) by viable cell count assay.

### 4.7. Statistical Analysis

All results are represented as the mean value ± standard deviation (SD) of three different experiments. The statistical analysis was performed using comparisons between groups evaluated by Student’s *t*-test or two-way ANOVA, followed by Bonferroni post hoc test using GraphPad Prism software (version 5; GraphPad Software, Inc., La Jolla, CA, USA), and only a value of *p* < 0.05 was considered statistically significant.

## 5. Conclusions

We succeeded in preparing both pristine inorganic magnetite (Fe_3_O_4_) core/organic nonpolar SLS-shell NPs and their functionalization with antimicrobial extra shells of UA and CEF, respectively. The prepared nanopowders were characterized from the physicochemical point of view by XRD, TEM, SAED, SEM, FTIR, and TGA combined with DSC. The optimal laser beam fluence was set at 300 mJ/cm^2^ by means of comparative analysis of the FTIR chemical maps of the coatings deposited at different fluence values. The MAPLE-deposited coatings showed excellent biocompatibility with eukaryotic cells, as demonstrated by the MTT cell viability test, LDH leakage assay, and NO inflammatory marker test performed on the MC3T3-E1 cell line as well as by monitoring the possible changes in cell morphology through fluorescence microscopy imaging. They also significantly inhibited the development of *S. aureus* and *P*. *aeruginosa* biofilms at 24 and 48 h. Thus, in the case of *S*. *aureus* strain, the Fe_3_O_4_@SLS/UA film reduced the number of CFU from 1 × 10^10^/mL for the control to a value a little bit higher than 1 × 10^5^ CFU/mL at 24 h and from 1 × 10^12^ for the control to a value less than 1 × 10^8^ CFU/mL at 48 h. Similarly, the Fe_3_O_4_@SLS/CEF coating reduced the number of CFU/mL from the control value to a value less than 1 × 10 ^6^CFU/mL at 24 h and to a value of 1 × 10^7^ CFU/mL at 48 h, respectively. In the case of the *P. aeruginosa* strain, at 24 h, the strongest inhibition effect was observed for the Fe_3_O_4_@SLS/UA sample, which reduced the number of CFU/mL from 1 × 10^11^ for the control to less than 1 × 10^7^ followed by Fe_3_O_4_@SLS/CEF (1 × 10^7^ CFU/mL) and Fe_3_O_4_@SLS, with a slight inhibition effect. At 48 h, the same trend of the inhibition effect was maintained. The most efficient coating was Fe_3_O_4_@SLS/UA, reducing the CFU/mL value from 1 × 10^12^ (control) to a value a little bit higher than 1 × 10^8^. The Fe_3_O_4_@SLS/CEF and Fe_3_O_4_@SLS also inhibited biofilm growth from the control value to 1 × 10^9^ CFU/mL and to 1 × 10^11^ CFU/mL, respectively. The above properties recommend our new magnetic nanopowders and MAPLE-deposited coatings as valuable candidates for future applications in the treatment of chronic and recurrent biofilm infections with these pathogenic bacteria.

## Data Availability

Data are contained within the article.
